# DCRDF-Net: A Dual-Channel Reverse-Distillation Fusion Network for 3D Industrial Anomaly Detection

**DOI:** 10.3390/s26020412

**Published:** 2026-01-08

**Authors:** Chunshui Wang, Jianbo Chen, Heng Zhang

**Affiliations:** 1 School of Computer and Information Science, College of Software, Southwest University, Chongqing 400715, China; wangchunshui@email.swu.edu.cn; 2 Kawasaki (Chongqing) Robotics Engineering Co., Ltd., Chongqing 400700, China; chenjianbo@kwskcq.com

**Keywords:** 3D anomaly detection, unsupervised learning, multimodal fusion

## Abstract

Industrial surface defect detection is essential for ensuring product quality, but real-world production lines often provide only a limited number of defective samples, making supervised training difficult. Multimodal anomaly detection with aligned RGB and depth data is a promising solution, yet existing fusion schemes tend to overlook modality-specific characteristics and cross-modal inconsistencies, so that defects visible in only one modality may be suppressed or diluted. In this work, we propose DCRDF-Net, a dual-channel reverse-distillation fusion network for unsupervised RGB–depth industrial anomaly detection. The framework learns modality-specific normal manifolds from nominal RGB and depth data and detects defects as deviations from these learned manifolds. It consists of three collaborative components: a Perlin-guided pseudo-anomaly generator that injects appearance–geometry-consistent perturbations into both modalities to enrich training signals; a dual-channel reverse-distillation architecture with guided feature refinement that denoises teacher features and constrains RGB and depth students towards clean, defect-free representations; and a cross-modal squeeze–excitation gated fusion module that adaptively combines RGB and depth anomaly evidence based on their reliability and agreement.Extensive experiments on the MVTec 3D-AD dataset show that DCRDF-Net achieves 97.1% image-level I-AUROC and 98.8% pixel-level PRO, surpassing current state-of-the-art multimodal methods on this benchmark.

## 1. Introduction

With the rapid advancement of industrial automation and intelligent manufacturing, precise detection of product surface defects has become a critical technology for ensuring production quality [1]. In real industrial environments, the vast majority of samples are normal, while anomalous ones are scarce, which makes Unsupervised Anomaly Detection (UAD) trained solely on normal samples a core direction in applied research [2]. RGB-based surface anomaly detection has seen significant progress, with the performance of many methods approaching its practical limit on the widely adopted MVTec AD benchmark [2]. However, RGB is sensitive to illumination variations and lacks three-dimensional geometric information, which limits its performance in defect scenarios dominated by 3D deformations such as undulations and material loss.

With the increasing prevalence of 3D sensors in industrial settings [3] and the release of the MVTec 3D-AD dataset [4], multimodal defect detection has emerged as an important trend that leverages complementary texture and geometric information to compensate for the limitations of unimodal methods. Early multimodal approaches largely followed a memory-bank paradigm (e.g., BTF, M3DM, CPMF),constructing a repository of normal features during training and performing anomaly discrimination through feature distance retrieval during inference [5,6]. Although these methods are robust, their reliance on substantial storage and computationally intensive distance matching hinders their deployment in resource-constrained industrial environments.

Current representative technical routes for multimodal industrial anomaly detection primarily encompass reconstruction-based methods and teacher–student distillation. Reconstruction-based methods typically perform self-supervised learning with synthetic anomalies and label anomalies via reconstruction residuals; however, they often overlook cross-modal joint denoising and consistency constraints, leading to anomaly restoration and background sensitivity, which, in complex scenes, cause false positives and false negatives. Teacher–student distillation adopts a frozen teacher to extract normal representations and trains a student to regress the teacher’s outputs; yet prior work has often employed isomorphic single-branch designs for the teacher and student, wherein fused representations tend to wash out modality-specific anomaly cues, causing cross-modal interference, insufficient anomaly contrast, and reduced separability for fine-grained defects. To address these issues, we propose DCRDF-Net (Dual-Channel Reverse-Distillation Fusion Network), which learns modality-specific normality manifolds on aligned RGB and 3D data and performs detection and localization through a unified deviation measure. Our main contributions can be summarized as follows:

- **Perlin-guided perturbation and texture matching:** In the foreground region, multi-frequency Perlin multiplicative perturbations driven by a shared random seed are synchronously applied to both color and depth data at corresponding spatial locations. For the RGB branch, textures are randomly sampled from the Describable Textures Dataset (DTD) for texture matching and statistically consistent fusion; for the depth branch, geometrically consistent guided smoothing is performed.
- **Dual-channel reverse distillation:** Reverse distillation is employed for both the RGB and depth branches, with a Guided Feature Denoising Network inserted between the teacher and student networks. This network suppresses defect-corrupted responses in the teacher’s outputs, ensuring the student network learns a pristine representation of normal samples.
- **Cross-modal squeeze–excitation gated fusion:** Channel recalibration leveraging cross-modal global statistics is integrated with depthwise-separable spatial gating. This framework learns pixel-wise weight maps based on the evidential strengths of the two student networks and their inter-modal discrepancies, thus achieving adaptive fusion of the two modalities.

## 2. Related Work

### 2.1. Two-Dimensional Industrial Anomaly Detection

Image anomaly detection aims to localize defects from a single RGB image of the target. The release of public datasets such as MVTec AD [2] and VisA [7] has substantially accelerated the development of related methods. Current mainstream approaches fall into three paradigms: reconstruction-based, normalizing-flow-based, and teacher–student distillation.

Reconstruction-based methods typically employ encoder-decoder architectures to reconstruct images or their intermediate features, using reconstruction error as the anomaly criterion. DRAEM [8] constructs a parallel architecture of reconstruction and discriminative subnetworks, simultaneously learning joint representations of anomalous images and their reconstruction results, as well as the classification boundary between them. To alleviate the coarse artifacts produced by autoencoder reconstructions, Liu et al. [9] proposed a two-stage pipeline that coordinates an Impression Extractor Network (IE-Net) with an expert network, which significantly improves the reconstruction fidelity of normal sample. Wolleb et al. [10] proposed a strategy within the diffusion model framework that combines iterative noise injection with classifier-guided image-to-image translation, enabling the generation of high-resolution anomaly heatmaps without requiring complex training. For low-light and complex illumination scenarios, some works have leveraged paired well-lit/low-light images for unsupervised learning, reconstructing well-lit features from low-light features and using the resulting inconsistency to identify anomalies, achieving superior detection and localization performance on datasets like LL-IAD [11].

Normalizing flow-based methods project the features of normal samples onto a tractable latent distribution through reversible affine transformations, and then determine the anomaly status of test samples based on their likelihood under the learned distribution. CFLOW-AD [12] integrated a pre-trained convolutional encoder with a multi-scale generative decoder under a conditional normalizing flow framework. It directly estimated the likelihood of encoded features, enabling real-time unsupervised anomaly detection and localization while maintaining memory and computational efficiency. CS-Flow [13] proposed a fully convolutional cross-scale normalizing flow architecture. Multi-scale feature maps were processed in parallel to capture both local and global context, and a spatial likelihood map was generated while preserving image structure, achieving interpretable and precise pixel-level defect localization. DifferNet [14] combined convolutional features with normalizing flows by first modeling the distribution of normal samples in a multi-scale feature space. Anomaly scores were then derived from pixel-wise likelihood differences, and robustness was improved through multiple image transformations.

Uninformed Students [15] introduced an end-to-end student–teacher distillation framework, in which a teacher network, pre-trained on large-scale natural images, extracted multi-scale local descriptors. The student network was trained to regress the teacher’s outputs, and a dense anomaly map was generated using prediction variance and regression error. Anomalies were detected when the student failed to accurately reproduce the teacher’s features on anomalous samples. IKD [16] tackled overfitting in distillation by introducing a Context Similarity Loss (CSL) to strengthen the perception of the normal data manifold structure, and employed Adaptive Hard Sample Mining (AHSM) to dynamically focus on informative challenging samples, thereby distilling more discriminative knowledge. RD++ [17] incorporated self-supervised optimal transport into a reverse distillation framework to improve the compactness of normal features. It also used simplex noise to generate pseudo-anomalous samples, reducing the student network’s sensitivity to anomalous signals and enhancing discrimination between normal and anomalous samples. In addition, for industrial surface segmentation and specific component inspection, NC-Net [18] improved pixel-level segmentation robustness via nested U-blocks and attention-based edge/mask guidance. In chip inspection scenarios, an efficient backbone combined with reverse feature matching and masking was used to achieve interpretable anomaly localization, supported by a real industrial dataset and ablation studies [19].

### 2.2. Three-Dimensional Industrial Anomaly Detection

#### 2.2.1. Memory Bank Methods

In 3D anomaly detection, memory-bank-based methods have become an established technique, which identifies anomalies by comparing input data against a stored repository of features from normal samples. M3DM [5] designed a multi-3D memory model that leveraged multiple memory banks together with a hybrid feature-fusion strategy to enhance cross-modal interaction and reduce information loss. CPMF [6] aggregated multimodal features and stored the fused representation in a single memory bank, reducing the memory footprint and improving inference speed. Shape-Guided [20] proposed a shape-guided expert-learning framework that constructed bimodal memory banks for expert training and linked them via correspondences, thereby fully exploiting geometric and appearance cues. M3DM-NR [21], building on M3DM, added a two-stage denoising network to process the inputs and replaced farthest-point sampling with an aligned multi-scale feature-extraction module, improving local feature extraction for point clouds. These methods require substantial memory to construct the banks, and the resulting memory pressure slows inference, making them ill-suited to real-time industrial defect-detection scenarios.

#### 2.2.2. Reconstruction-Based Methods

Reconstruction-based methods typically learn, on normal data, to self-supervise the reconstruction of the input modalities (RGB images and depth maps), and use the reconstruction error as the anomaly criterion. EasyNet [22] designed a dual-branch multimodal reconstruction network that independently restored RGB and depth inputs and used an information-entropy comparison strategy to decide whether to fuse depth features, achieving fast inference and deployment practicality. DBRN [23] likewise adopted a dual-branch reconstruction architecture, introduced Perlin noise on depth maps to generate anomaly masks, used grayscale images from natural-texture datasets to simulate realistic defect textures, and designed an importance-scoring module to assess and fuse the two modalities. 3DSR [24] proposed a Depth-Aware Discrete Autoencoder (DADA) that jointly modeled RGB and 3D data in a discrete latent space, improving the representation and detection of 3D surface anomalies. Uni-3DAD [25] proposed an architecture with a feature-based branch and a reconstruction-based branch to detect surface defects and missing regions, and finally fused the two outputs using an OCSVM. These approaches often prioritized self-supervised reconstruction while neglecting cross-modal joint denoising and fusion strategies, which led to false positives and false negatives in complex scenes.

#### 2.2.3. Teacher–Student Methods

In multimodal anomaly detection, the teacher–student paradigm leverages the teacher’s strong representation of normal samples to guide the student; when a test sample deviates from normal patterns, the discrepancy between student and teacher outputs serves as an anomaly cue. AST [26] adopted an asymmetric teacher–student design, using a conditional normalizing flow as the teacher and a convolutional network as the student, and fed concatenated RGB and depth features into the model; by exploiting the bijective property of flows, it further amplified the teacher’s output disparity between normal and abnormal samples, thereby strengthening the student’s anomaly response. MMRD [27] proposed a multimodal reverse-distillation approach: on the teacher side, twin encoders extracted RGB and depth features in parallel to enable parameter-free fusion, whereas on the learnable student decoder side, cross-modal interaction and reconstruction-error computation drove the student to more accurately recover the distribution of normal samples. LPFSTNet [28] designed, for 3D industrial scenarios, a lightweight, parameter-free “head-attention” teacher–student network: the student comprised only simplified convolutions, reducing parameters and accentuating the architectural gap from the teacher to improve generalization. Existing methods commonly suffer from representation homogenization due to isomorphic teacher–student pairs and from anomaly-signal smoothing caused by single-branch fusion.

Across these three lines of work, a common limitation is that RGB and depth are either coupled too early or fused in a heuristic manner. Memory-bank methods incur substantial storage and retrieval overhead and typically treat fused features as static templates. Reconstruction-based models emphasize self-supervised restoration but rarely perform joint cross-modal denoising, which makes them prone to restoring anomalies and amplifying background clutter. Teacher–student approaches often rely on isomorphic, single-branch designs, so that fusion tends to homogenize modality-specific cues and smooth out fine-grained anomaly signals. In contrast, DCRDF-Net is explicitly designed to (i) avoid external memory banks and learn modality-specific normality manifolds under a shared frozen teacher via dual-channel reverse distillation, (ii) enrich supervision with a Perlin-guided pseudo-anomaly generator that injects appearance–geometry-consistent perturbations at aligned RGB–depth locations, and (iii) perform adaptive, pixel-wise fusion through a cross-modal squeeze–excitation gated module that exploits both unimodal evidence and cross-modal discrepancy.

## 3. Method

### 3.1. Overview of the Methodology

**Figure 1:**
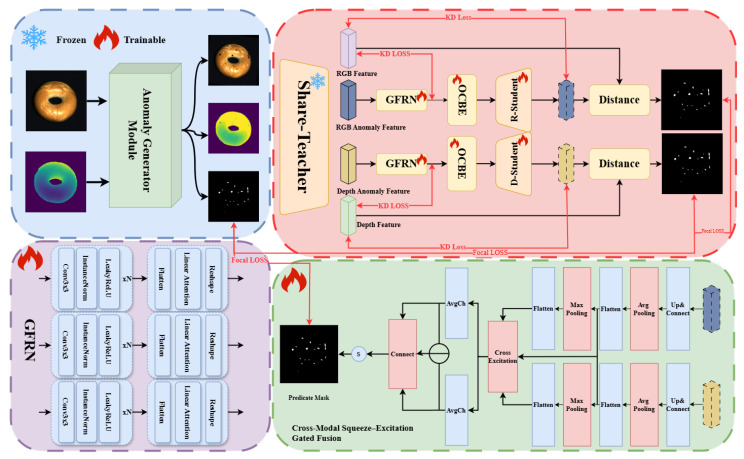
Overall architecture of the proposed DCRDF-Net.

The overall pipeline and key module designs of the proposed DCRDF-Net are shown in Figure 1. The method begins by synchronously injecting appearance-geometry-consistent pseudo-anomalies into registered RGB-Depth pairs. Specifically, for the RGB modality, we employ multi-frequency Perlin noise—statistically aligned with external textures—to induce foreground-aware and boundary-preserving perturbations. Concurrently, structurally consistent geometric perturbations are applied to the depth channel via proportional scaling. In the feature-learning stage, a shared teacher encoder, coupled with a Guided Feature Refinement Network (GFRN), performs projection-based compression and linear-attention refinement on the perturbed multi-scale features, thereby recovering clean representations that closely approximate the defect-free prior. Subsequently, two independent student branches conduct reverse reconstruction for the RGB and depth modalities, respectively, to obtain within-modality anomaly response maps. Finally, a cross-modal squeeze–excitation gated fusion module is constructed, which leverages global channel priors and depthwise-separable spatial gating to generate pixel-wise adaptive weights.

The construction strategy for the consistency-preserving pseudo-anomalies is detailed in Section 3.2. Section 3.3 then elaborates on the teacher–student reverse-distillation network and the Guided Feature Refinement Network (GFRN). Finally, the design of the cross-modal gated fusion network is described in Section 3.4.

### 3.2. Perlin-Guided Percentage-Based Perturbation and Statistical Texture Alignment

To synchronously inject appearance–geometry–consistent pseudo anomalies at identical pixel coordinates, we denote the aligned RGB image and depth map by *R* and *D*, respectively. We first construct a soft-foreground mask $M_{s}$ from a binary foreground $M_{f}$ with boundary attenuation, where the edge-decay weight $w_{\mathrm{edge}}$ depends exponentially on the distance to the foreground boundary $\mathrm{dist}(M_{f})$, τ is the decay scale, and α is the softening coefficient:

(1) $$w_{\mathrm{edge}}\left(x,y\right)\;=\;\exp\;\left(-\frac{\mathrm{dist}\left(M_{f}\right)\left(x,y\right)}{\tau }\right).$$

(2) $$M_{s}\left(x,y\right)\;=\;\min\;\left(1,\;M_{f}\left(x,y\right)+\alpha \;w_{\mathrm{edge}}\left(x,y\right)\right).$$

Concretely, $\mathrm{dist}(M_{f})$ is computed using the standard Euclidean distance transform on the binary foreground mask $M_{f}$, yielding for each pixel its Euclidean distance to the nearest foreground boundary. This distance is then normalized by the maximum distance inside the foreground region so that it lies in [0, 1], and subsequently used in Equation (1) to generate the edge-decay weight $w_{\mathrm{edge}}$.

The texture intensity *T* is obtained from luminance gradients and local variance and is mapped to a normalized weight $\hat{T}$ via a sigmoid. Using a single random seed *s*, we generate three-frequency multi-scale Perlin noise with amplitude compression, where $P(f_{i};s)$ is the 2D Perlin field at frequency $f_{i}$, $\eta_{i}$ are mixture weights, and κ is the compression coefficient:

(3) $$\tilde{P}\;=\;\tanh\;\left(\kappa \sum_{i=1}^{3}\eta_{i}\;P\left(f_{i};s\right)\right).$$

In practice, for 256 × 256 inputs we generate Perlin fields on 16 × 16, 32 × 32, and 64 × 64 grids, corresponding to three frequencies $f_{1},f_{2},f_{3}$ that cover coarse-, mid-, and fine-scale structures in the foreground. The mixture weights are fixed to $\left(\eta_{1},\eta_{2},\eta_{3}\right)=\left(0.5,\;0.3,\;0.2\right)$ to emphasize mid-scale components while suppressing excessive high-frequency noise, and the compression coefficient is set to κ=1.0. After the tanh(·) nonlinearity, $\tilde{P}$ remains in a moderate range and provides sufficient contrast for the percentage-based modulation in Equations (6) and (7). These hyperparameters are selected once on a small held-out subset and kept fixed for all categories and experiments on the MVTec 3D-AD dataset.

Within the foreground, we construct a per-pixel probability map *U* from the structure field, texture weight, and boundary weight; here σ(·) is the sigmoid, θ is a quantile threshold, ⊮[·] is the indicator function, and ${\mathrm{Close}}_{3\times 3}\left(\cdot \right)$ denotes morphological closing. The final mask *M* is given by

(4) $$U\;=\;\sigma \;\left(\tilde{P}\cdot \hat{T}\cdot w_{\mathrm{edge}}\right),$$

(5) $$M\;=\;{\mathrm{Close}}_{3\times 3}\;\left(M_{s}\cdot ⊮\left[\;U>\theta \;\right]\right).$$

For the appearance branch, we select a texture image *N* from the DTD library and align it via affine transformation and channel-statistics matching to obtain $\hat{N}$, which matches the foreground statistics of *R*. Concretely, we compute the tight bounding box of $M_{s}$ and resize/crop the sampled DTD texture to this box with a small random scale and rotation, yielding an affine mapping from the DTD coordinates to the image plane. We then match the per-channel mean and standard deviation of the transformed texture to those of *R* within the foreground region so that $\hat{N}$ is visually consistent with the object appearance before blending. Let the pixel transparency ρ(x,y) be determined by texture and gradient cues, and define the foreground modulation field $S_{f}=M\cdot w_{\mathrm{edge}}\cdot \hat{T}$. With percentage amplitude $\beta_{p}$ for color channels, element-wise/channel-wise product ⊙, and intensity clipping clip(·), we compute

(6) $$R^{a}\;=\;\left(1-M\right)⊙R\;+\;M⊙\;\left(\left(1-\rho \right)⊙\mathrm{clip}\;\left(R⊙(1+\beta_{p}\;S_{f}\;\tilde{P})\right)\;+\;\rho ⊙\hat{N}\right),$$

where α in Equation (2) is the softening coefficient for $M_{s}$, while ρ(x,y) here denotes the per-pixel transparency. This procedure ensures that the injected texture is spatially aligned with the object region and smoothly blended with the original appearance according to the mask *M* and transparency map ρ(x,y).

For the geometry branch, we maintain consistency with the surface structure. We inpaint to obtain $D_{\mathrm{fill}}$ and guided-smooth $\tilde{P}$ with $D_{\mathrm{fill}}$ to produce ${\tilde{P}}_{d}$. With depth-channel percentage amplitude $\beta_{d}$, the perturbed depth is

(7) $$D^{a}\;=\;\left(1-M\right)\cdot D_{\mathrm{fill}}\;+\;M\cdot D_{\mathrm{fill}}\cdot \left(1+\beta_{d}\;S_{f}\;{\tilde{P}}_{d}\right),$$

where $R^{a}$ and $D^{a}$ denote the RGB image and depth map after pseudo-anomaly injection, and *M* is the final mask produced by the generation process.

### 3.3. Dual-Channel Reverse-Distillation Network

Given the heterogeneity of defect representations across modalities, RGB images are more sensitive to color and luminance variations and can precisely capture subtle changes of this kind, whereas depth maps possess stronger discrimination for geometric relief and surface micro-topography, effectively characterizing related structural attributes. Early fusion at shallow network layers tends to cause fusion instability, whereby one modality dominates and suppresses the other. Accordingly, we adopt branch-wise independent modeling with decision-level fusion: first, we construct a dual-channel reverse-distillation network, enabling the RGB and depth branches to perform feature denoising, modality alignment, and defect detection within their respective channels; then, at the decision stage, we introduce a cross-modal squeeze–excitation gated fusion module that achieves adaptive weighted fusion via global channel statistics and pixel-wise evidence analysis. In contrast to classic teacher–student pipelines that jointly adapt the teacher on the target domain, we keep the teacher frozen and only update the denoising and student branches so that the teacher acts as a stable cross-modal semantic prior and the teacher–student discrepancy remains sensitive to local anomalies.

#### 3.3.1. Teacher Encoder

We employ a ResNet-50 backbone, pre-trained on ImageNet and kept frozen throughout training, as the shared teacher encoder Φ for both modalities, serving as a modality-agnostic, structure-sensitive prior rather than being fine-tuned on the target dataset. The original RGB image *R* and depth map *D* (where *D* is first normalized and replicated along the channel dimension to form a three-channel “pseudo-RGB” input that matches the ResNet-50 input format), together with their Perlin-perturbed counterparts $R^{a}$ and $D^{a}$, are processed by the teacher encoder to extract multi-scale features ${\left\{F_{i}^{0},D_{i}^{0},F_{i}^{a},D_{i}^{a}\right\}}_{i=1}^{3}$; the clean features $\left\{F_{i}^{0},D_{i}^{0}\right\}$ provide direct supervision targets for both the guided feature denoising network and the student network while also defining a common semantic coordinate system shared by the RGB and depth branches.

#### 3.3.2. Guided Feature Refinement Network

The guided feature refinement network (GFRN) aims to recover the teacher’s defect-free multi-scale representations, aligning the student-side pyramid with $\left\{F_{i}^{0},D_{i}^{0}\right\}$ in distribution while preserving sharp boundaries and fine details. At each scale, a stack of projection layers performs constrained denoising and distribution contraction (channel recalibration and instance normalization), pushing anomaly-induced first-order responses back onto the teacher’s low-variance submanifold and yielding clean, prior-centered features. Concretely, each projection stack is implemented as two consecutive 3 × 3 convolutional blocks, where each block consists of a 3 × 3 convolution followed by InstanceNorm and a LeakyReLU activation; this design focuses on locally suppressing anomaly-induced noise and shrinking the feature distribution toward the normal teacher manifold. We then use linear attentionas a non-local refiner to aggregate long-range self-similarity at the same spatial resolution; a position-aware bias and residual write-back suppress cross-boundary leakage and over-smoothing, preserving steep boundary transitions. In practice, we adopt a lightweight linear-attention block that directly operates on the feature map and is wrapped in a residual connection, so that global consistency is enhanced without introducing additional downsampling or heavy transformer layers. The purified features are denoted by ${\tilde{F}}_{i}$ and ${\tilde{D}}_{i}$. Projection layers handle local denoising and variance reduction, whereas linear attention enforces global consistency and boundary fidelity. We instantiate two GFRNs with identical architecture but independent parameters for the RGB and depth branches, allowing each modality to adapt to its own statistics; for the depth branch, the GFRN operates on features extracted from the replicated and normalized three-channel depth input, so that stable modality-specific biases in the frozen teacher responses are implicitly absorbed by the refinement network and the depth-side student, while the remaining teacher–student discrepancy primarily reflects anomaly-induced deviations rather than deterministic cross-modal differences.

Training minimizes a scale-wise denoising-distillation loss that directly matches the denoised outputs to the teacher’s clean features:

(8) $$\begin{array}{ccc} L_{\mathrm{denoise}}\;=\;\; & \sum_{i=1}^{3}∥{\tilde{F}}_{i}-F_{i}^{0}{∥}_{2}^{2}\; & \;+\;\sum_{i=1}^{3}∥{\tilde{D}}_{i}-D_{i}^{0}{∥}_{2}^{2}\;. \end{array}$$

#### 3.3.3. Student Decoder

A ResNet-50 decoder reconstructs each modality branch. The denoised features ${\left\{{\tilde{F}}_{i},{\tilde{D}}_{i}\right\}}_{i=1}^{3}$ first pass through a lightweight OCBE (Output Calibration and Boundary Enhancement) module for distribution regularization and bandwidth control, then are decoded to produce student reconstructions ${\left\{F_{i}^{S},D_{i}^{S}\right\}}_{i=1}^{3}$.

We compute multi-scale distances between the student’s reconstructed features and the teacher’s pristine features $\left\{F_{i}^{0},D_{i}^{0}\right\}$, followed by upsampling and aggregation to obtain per-modality anomaly prediction maps $p_{r}$ and $p_{d}$. To inherit the teacher’s soft distribution while achieving pixel-level alignment with anomaly masks, we employ two complementary losses: feature-level knowledge distillation $L_{\mathrm{KD}}$ for channel-distribution and confidence-shape alignment, and pixel-level focal loss $L_{\mathrm{focal}}$ to handle class imbalance and improve small-defect recall.

The overall student objective is:

(9) $$L_{\mathrm{stu}}\;=\;\lambda_{\mathrm{KD}}\;L_{\mathrm{KD}}\;+\;\lambda_{\mathrm{seg}}\;L_{\mathrm{focal}},$$

where $\lambda_{\mathrm{KD}},\lambda_{\mathrm{seg}}\geq 0$ (both set to 1 during training).

### 3.4. Cross-Modal Squeeze–Excitation Gated Fusion

We construct a cross-modal squeeze–excitation gated fusion (CM-SEGF) module that generates pixel-wise adaptive weights via global channel recalibration and depthwise-separable spatial gating, enabling calibrated fusion of the two modality predictions. First, multi-scale features from the two student branches are upsampled and concatenated to obtain $F_{r},F_{d}\;\in \;R^{B\times C\times H\times W}$. For each modality, global average/max pooling yields $a_{r},m_{r},a_{d},m_{d}\;\in \;R^{B\times C}$. We form cross-modal descriptors $z_{r}=\left[a_{r},m_{r},a_{d},m_{d}\right]$ and $z_{d}=\left[a_{d},m_{d},a_{r},m_{r}\right]$ and map them through an MLP to channel gates $g_{r},g_{d}\;\in \;{\left[0,\;1\right]}^{C}$, then recalibrate features via

(10) $${\tilde{F}}_{m}\;=\;g_{m}⊙F_{m},\;m\in \left\{r,d\right\}.$$

From the recalibrated features we extract three single-channel cues—RGB evidence $E_{r}=\mathrm{AvgCh}(|{\tilde{F}}_{r}\left|\right)$, depth evidence $E_{d}=\mathrm{AvgCh}(|{\tilde{F}}_{d}\left|\right)$, and cross-modal discrepancy $\Delta_{H}=\mathrm{AvgCh}(|{\tilde{F}}_{r}-{\tilde{F}}_{d}\left|\right)$—and stack them as $X=\left[E_{r},E_{d},\Delta_{H}\right]\;\in \;R^{B\times 3\times H\times W}$. A depthwise-separable block models spatial context and mixes channels to produce a pre-activation, and the pixel-wise weight field is

(11) $$s\;=\;\sigma \;\left(\mathrm{DSConv}\left(\left[\;\mathrm{AvgCh}(\right|{\tilde{F}}_{r}\left|),\;\mathrm{AvgCh}(\right|{\tilde{F}}_{d}\left|),\;\mathrm{AvgCh}(\right|{\tilde{F}}_{r}-{\tilde{F}}_{d}\left|)\;\right]\right)\right)\;\in \;{\left[0,\;1\right]}^{B\times 1\times H\times W}.$$

The fused prediction explicitly balances the two modalities:

(12) $$p_{\mathrm{fused}}\;=\;s\;p_{r}\;+\;\left(1-s\right)\;p_{d},$$

where $p_{r},p_{d}\;\in \;\left[0,\;1\right]$ are pixel-level predictions from the RGB and depth branches, respectively. The fusion objective uses Focal Loss as the sole supervision:

(13) $$L_{\mathrm{fused}}\;=\;\lambda_{\mathrm{fused}}\;L_{\mathrm{focal}}\;\left(p_{\mathrm{fused}},\;M\right),\;\lambda_{\mathrm{fused}}=1.$$

## 4. Experimental

### 4.1. Experimental Details

**Dataset:** We conducted experiments on the MVTec 3D-AD dataset [4], a 3D industrial anomaly-detection benchmark comprising 10 object categories. The training set contains 2656 normal samples, each consisting of an RGB image and the corresponding 3D scan information. The test set includes 1197 samples—948 anomalous and 249 normal—and provides pixel-level annotations for evaluating both detection and localization. In addition, MVTec 3D-AD provides a validation set with 294 normal samples.

**Implementation Details:** We used co-registered 256 × 256 RGB–depth pairs as inputs for both training and inference. During training, we synthesized geometrically conformal pseudo anomalies within foreground regions using a Perlin-based, texture-aligned strategy to mimic real defect distributions; at inference, raw image pairs were fed directly. The backbone was ResNet-50, and models were trained and evaluated independently per category. We optimized with Adam (learning rate 0.005, batch size 16) for a total of 150 epochs. All experiments were conducted on a single NVIDIA RTX 2080. (NVIDIA, Santa Clara, CA, USA) At inference, the network outputs anomaly heatmaps for the RGB branch, the depth branch, and their fused result.

**Evaluation Metrics:** To comprehensively assess the proposed method’s detection and localization performance, we adhere to the evaluation protocol established in recent studies on MVTec 3D-AD and multimodal industrial anomaly detection and use two standard metrics at the image and pixel levels. At the image level, we compute the image-level area under the ROC curve (I-AUROC) over all test samples to evaluate the model’s ability to recognize anomalous objects. I-AUROC is a threshold-independent metric that is relatively insensitive to class imbalance and has been widely adopted as the standard indicator for reporting image-level detection performance in industrial anomaly detection benchmarks. At the pixel level, we report the Per-Region Overlap (PRO) to quantify the model’s spatial accuracy in localizing anomalous regions. In contrast to purely pixel-counting measures, PRO aggregates the overlap over connected components of the ground-truth mask and is therefore more sensitive to the spatial continuity and completeness of detected defect regions.

### 4.2. Experimental Results and Analysis

**Figure 2:**
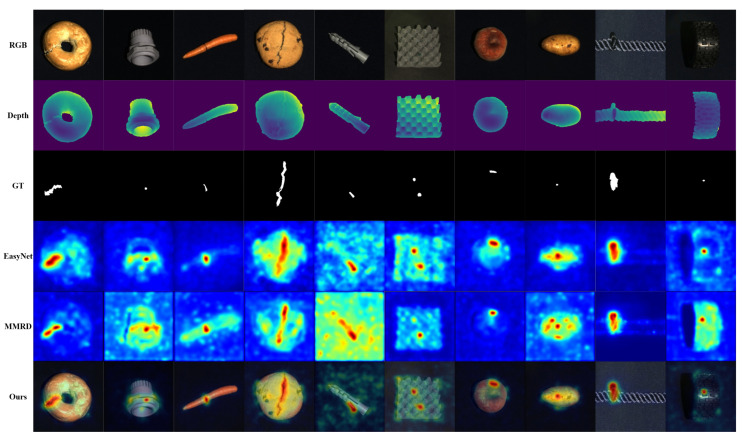
Qualitative comparison of anomaly localization on the MVTec 3D-AD dataset. The compared methods are shown using our implementations, and the last column presents the predictions of DCRDF-Net.

Table 1 and Table 2 report comparisons of image-level detection (I-AUROC) and pixel-level localization (PRO) on the MVTec 3D-AD dataset.Qualitative visualizations are shown in Figure 2. Overall, the proposed method leads on both metrics: the class-wise mean I-AUROC reaches 97.1% and the class-wise mean PRO reaches 98.8%, attaining best or near-best scores on most categories and exhibiting stable, well-balanced detection–localization performance.

**Image-level detection (I-AUROC):** As shown in Table 1, our method achieves the highest I-AUROC on Carrot, Cookie, Foam, and Tire (99.5%, 99.3%, 98.4%, and 92.8%, respectively), while other categories remain in a high range (e.g., Bagel 99.1%, Rope 98.1%), yielding an overall mean of 97.1%. Compared with 3D methods using only point clouds or depth (e.g., DepthGAN/AE/VM), our I-AUROC improves markedly across categories; relative to representative multimodal baselines (BTF, AST, M3DM, MMRD), our approach delivers strong results on both texture-dominated (Carrot, Cookie) and geometry-dominated (Foam, Tire) targets, indicating superior adaptability to heterogeneous defect patterns. A closer comparison shows that, for Foam/Tire, gains over single-modality or statically fused models stem from a more sensitive response to geometric undulations and local concavities; for Carrot/Cookie, compared with models that reconstruct only in the pixel or depth domain, our method achieves higher separability for fine-grained texture anomalies.

**Pixel-level localization (PRO):** As shown in Table 2, our method attains the highest PRO on Bagel, Carrot, Cookie, and Rope (99.1%, 99.5%, 98.5%,98.8%, respectively) and ranks among the top for most other categories, resulting in a mean of 98.8%, surpassing representative methods (e.g., 97.6% for MMRD, 97.6% for Shape-Guided, and 96.4% for M3DM). Notably, in scenes with complex textures (Foam, Peach) and slender structures (Rope, Cable Gland), our approach maintains high recall while effectively suppressing false positives, producing smoother anomaly heatmaps with sharper boundaries. These results indicate that the proposed denoising distillation and adaptive fusion preserve structural consistency while emphasizing anomalous details, thereby stabilizing localization metrics across diverse object morphologies.

**Runtime efficiency:** We further evaluated the runtime characteristics of DCRDF-Net on an experimental platform equipped with a single NVIDIA RTX 2080 GPU (12 GB) using a PyTorch (version 2.4.1) implementation. For aligned RGB–depth pairs of size 256 × 256 with a batch size of 1, the model attains an inference throughput of about 3 FPS, with a peak GPU memory footprint of around 3.2 GB during testing. Owing to the frozen teacher backbone, lightweight dual student branches, and the absence of external memory banks or heavy generative modules, the overall computational cost remains compatible with typical cycle-time requirements in in-line industrial inspection.

### 4.3. Ablation Study

To quantify the contribution of individual modalities and validate bimodal synergy, we evaluated three configurations under identical experimental conditions: using only depth (3D-only), only RGB, and both modalities (RGB+3D). As shown in Table 1 and Table 3, the class-wise mean I-AUROC for the three settings was 89.1%, 90.3%, and 97.1%, respectively; the bimodal setting achieved a clear and consistent improvement over either unimodal counterpart and exhibited stable advantages across categories. Per-category analysis indicated that texture-dominated categories (e.g., Carrot, Cookie) benefited markedly from RGB data, whereas geometry-dominated ones (e.g., Foam, Rope, Tire) showed substantially improved anomaly separability when depth information was incorporated. When the two modalities were combined, their advantages compounded—for example, Foam increased from 88.9% (3D-only) and 91.3% (RGB-only) to 98.4%; Tire rose from 83.3%/87.0% to 92.8%; and Carrot improved from 92.0%/92.4% to 99.5%.These results stem from cross-modal complementarity and consistency constraints: RGB is highly sensitive to fine-grained textures and color changes, whereas depth offers stronger structural discrimination for deformations, surface undulations, and missing regions. Joint evaluation suppresses unimodal false alarms in texture-complex or cluttered scenes and compensates for RGB omissions in geometry-dominated cases, thereby systematically widening the distribution gap between normal and anomalous samples.

**Figure 3:**
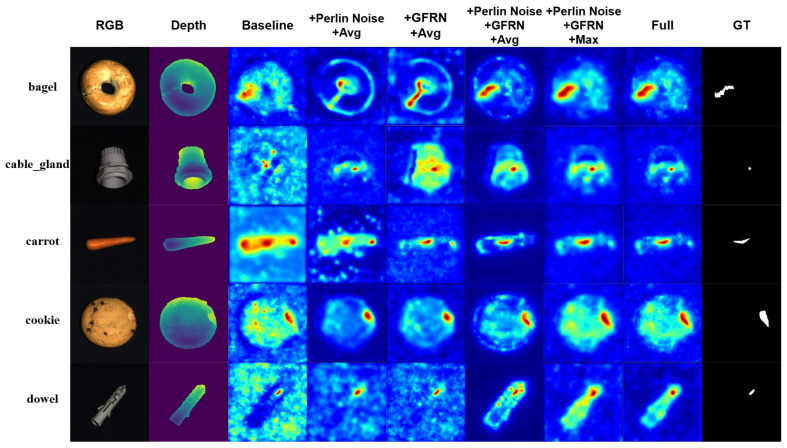
Qualitative comparison of anomaly heatmaps for different ablation variants of DCRDF-Net on the MVTec 3D-AD dataset.

As summarized in Table 4 and visualized in Figure 3, the baseline model, based on a ResNet50 teacher–student architecture, demonstrated limited discriminative capability for subtle defects in foreground regions and produced relatively diffuse, noisy anomaly responses in the background. With Perlin-guided percentage perturbations and texture/geometry alignment, the training signal became more challenging; normal–abnormal separability improved and the model’s reliance on background cues decreased markedly, which is reflected in Figure 3 by more concentrated responses around true defect regions. Incorporating the Guided Feature Refinement Network (GFRN) alone purified multi-scale features, enhanced long-range consistency and boundary fidelity, and widened the normal–abnormal margin, leading to cleaner and sharper anomaly maps. When both components were enabled, their synergy—stronger training supervision coupled with cleaner representations—yielded steady gains across metrics and visibly less cluttered heatmaps in Figure 3.

Fusion strategies. As also illustrated in Figure 3, max fusion generally delivered higher sensitivity than equal-weight averaging but produced isolated high-response regions that impaired spatial continuity. By contrast, the proposed Cross-Modal Squeeze–Excitation Gated Fusion (CM-SEGF) leveraged student semantic features to recalibrate channels and perform pixel-wise adaptive weighting, so that modality contributions are increased near true defects and suppressed in background regions, resulting in more contiguous and accurate anomaly maps in Figure 3 and thereby achieving more robust performance at both the image and pixel levels.

## 5. Conclusions

We have presented DCRDF-Net, which achieves robust multimodal unsupervised industrial anomaly detection through three synergistic designs: (i) a consistency-preserving pseudo-anomaly generator that synchronously injects conformal perturbations into appearance and depth within the foreground via multi-frequency Perlin guidance and texture/geometry alignment, yielding position-controllable and difficulty-adjustable training signals; (ii) a Guided Feature Refinement Network (GFRN) that aligns the frozen teacher’s defect-free prior on the student side via multi-layer projection compression and linear attention, purifying multi-scale representations while preserving boundary fidelity; and (iii) Cross-Modal Squeeze–Excitation Gated Fusion (CM-SEGF), which generates pixel-wise weights from both channel and spatial perspectives based on student semantics, explicitly calibrating the RGB/depth contributions and improving regional consistency and robustness. Experiments demonstrated the proposed model’s state-of-the-art performance on MVTec 3D-AD.

Although DCRDF-Net achieves strong detection and localization performance on MVTec 3D-AD, it still has several limitations. First, the Perlin-guided pseudo anomalies cover only a restricted family of defect morphologies and scales and are not intended to visually mimic the full diversity of real industrial defects. In particular, we observe that for a small subset of samples with large-scale structural damage or extremely specular surfaces the synthetic perturbations provide weaker guidance, and the performance margin over strong baselines becomes smaller. Second, the current study assumes that RGB and depth inputs are reasonably well-formed and does not yet systematically evaluate robustness under severely corrupted or missing single-modality conditions (e.g., heavily saturated or highly noisy depth channels). In addition, we reused an ImageNet-pretrained RGB encoder as a frozen teacher for the depth modality via channel replication. While this design works well under our experimental setup, it may still leave room for improved domain adaptation in more complex or strongly geometry-dominated depth scenarios. Future work will therefore focus on enriching the pseudo-anomaly distribution with more physically grounded manufacturing priors, as well as on exploring depth-aware or multimodal pre-training and more robust designs for handling extreme single-modality degradation.

## Figures and Tables

**Table 1:** Image-level anomaly detection results (I-AUROC%) on MVTec 3D-AD. (bold values denote the maximum values of each column).

Method	Bagel	Cable Gland	Carrot	Cookie	Dowel	Foam	Peach	Potato	Rope	Tire	Mean
DepthGAN	53.8	37.2	58.0	60.3	43.0	53.4	64.2	60.1	44.3	57.7	53.2
DepthAE	64.8	50.2	65.0	48.8	80.5	52.2	71.2	52.9	54.0	55.2	59.5
DepthVM	51.3	55.1	47.7	58.1	61.7	71.6	45.0	42.1	59.8	62.3	55.5
BTF	91.8	74.8	96.7	88.3	93.2	58.2	89.6	91.2	92.1	88.6	86.5
Shape-guided	98.6	89.4	98.3	99.1	97.6	85.7	**99.0**	96.5	96.0	86.9	94.7
AST	98.3	87.3	97.6	97.1	93.2	88.5	97.4	**98.1**	**100.0**	79.7	93.7
EasyNet	99.1	**99.8**	91.8	96.8	94.5	94.5	90.5	80.7	99.4	79.3	92.6
M3DM	99.4	90.9	97.2	97.6	96.0	94.2	97.3	88.9	97.2	85.0	94.5
MMRD	**99.9**	94.3	96.4	94.3	**99.2**	91.2	94.9	90.1	99.4	90.1	95.0
**Ours**	99.1	98.9	**99.5**	**99.3**	96.2	**98.4**	95.1	93.7	98.1	**92.8**	**97.1**

**Table 2:** Pixel-level anomaly localization results (PRO%) on MVTec 3D-AD. (bold values denote the maximum values of each column).

Method	Bagel	Cable Gland	Carrot	Cookie	Dowel	Foam	Peach	Potato	Rope	Tire	Mean
DepthGAN	42.1	42.2	77.8	69.6	49.4	25.2	28.5	36.2	40.2	63.1	47.4
DepthAE	43.2	15.8	80.8	49.1	84.1	40.6	26.2	21.6	71.6	47.8	48.1
DepthVM	38.8	32.1	19.4	57.0	40.8	28.2	24.4	34.9	26.8	33.1	33.5
BTF	97.6	96.9	97.9	97.3	93.3	88.8	97.5	98.1	95.0	97.1	95.9
Shape-guided	98.1	97.3	98.2	97.1	96.2	97.8	98.1	98.3	97.4	97.5	97.6
AST	97.0	94.7	98.1	93.9	91.3	90.6	97.9	98.2	88.9	94.0	94.4
EasyNet	93.5	94.1	97.1	89.7	88.5	**99.7**	**99.2**	88.8	95.5	72.8	91.9
M3DM	97.0	97.1	97.9	95.0	94.1	93.2	97.7	97.1	97.1	97.5	96.4
MMRD	98.6	**99.0**	99.1	95.1	**99.0**	90.1	94.9	**99.0**	98.7	**98.2**	97.6
**Ours**	**99.1**	98.9	**99.5**	**98.5**	96.2	98.4	96.7	95.7	**98.8**	95.8	**98.8**

**Table 3:** I-AUROC(%) score for anomaly detection of MVTec 3D-AD. (bold values denote the maximum values of each column).

Group	Method	Bagel	Cable Gland	Carrot	Cookie	Dowel	Foam	Peach	Potato	Rope	Tire	Mean
3D	DepthGAN	53.0	37.6	60.7	60.3	49.7	48.4	59.5	48.9	53.6	52.1	52.3
	DepthAE	46.8	73.1	49.7	67.3	53.4	41.7	48.5	54.9	56.4	54.6	54.6
	FPFH	82.5	55.1	95.2	79.7	88.3	58.2	75.8	88.9	92.9	65.3	78.2
	3D-ST	86.2	48.4	83.2	89.4	84.8	66.3	76.3	68.7	95.8	48.6	74.8
	Shape-guided	**98.3**	68.2	**97.8**	**99.8**	96.0	73.7	**99.3**	**97.9**	96.6	**87.1**	**91.6**
	AST	88.1	57.6	96.5	95.7	67.9	79.7	99.0	91.5	95.6	61.1	83.3
	M3DM	94.1	65.1	96.5	96.9	90.5	76.0	88.0	97.4	92.6	76.5	87.4
	EasyNet	73.5	67.8	74.7	86.4	71.9	71.6	71.3	72.5	88.5	68.7	74.7
	MMRD	82.9	66.6	93.7	80.4	**97.2**	86.5	94.7	80.6	**96.7**	84.9	86.6
	**Ours**	92.6	**89.4**	92.0	91.8	87.7	**88.9**	87.6	87.2	90.6	83.3	89.1
RGB	PatchCore	87.6	88.0	79.1	68.2	91.2	70.1	69.5	61.8	84.1	70.2	77.0
	Shape-guided	91.1	93.6	88.3	66.2	97.4	77.2	78.5	64.1	88.4	70.6	81.5
	AST	94.7	92.8	85.1	82.5	**98.1**	**95.1**	89.5	61.3	**99.2**	82.1	88.0
	EasyNet	98.2	**99.2**	91.7	**95.3**	91.9	92.3	84.0	78.5	98.6	74.2	**90.4**
	M3DM	94.4	91.8	89.6	74.9	95.9	76.7	**91.9**	64.8	93.8	76.7	85.0
	MMRD	**98.7**	93.7	**94.3**	77.0	**98.1**	84.7	91.3	75.3	**99.3**	85.3	89.8
	**Ours**	92.0	91.8	92.4	92.2	89.1	91.3	89.0	**86.6**	92.0	**87.0**	90.3

**Table 4:** Architecture ablation on MVTec 3D-AD (I-AUROC and PRO, %). (bold values denote the maximum values of each column).

Variant	Perlin Noise	GFRN	Fusion	I-AUROC	PRO
Baseline	No	No	Avg	83.7	76.4
+ Perlin Noise	Yes	No	Avg	86.1	88.2
+ GFRN	No	Yes	Avg	86.5	75.7
+ Perlin Noise + GFRN (Avg)	Yes	Yes	Avg	88.4	80.4
+ Perlin Noise + GFRN (Max)	Yes	Yes	Max	89.1	90.6
**Full**	Yes	Yes	CM-SEGF	**97.1**	**98.8**

## Data Availability

Data supporting the findings of this study are available as follows: the MVTec 3D-AD benchmark dataset is publicly available at https://www.mvtec.com/company/research/datasets/mvtec-3d-ad (accessed on 4 January 2026) (subject to the dataset’s terms of use).

## References

[1] Lin Y., Chang Y., Tong X., Yu J., Liotta A., Huang G., Song W., Zeng D., Wu Z., Wang Y. (2025). A survey on RGB, 3D, and multimodal approaches for unsupervised industrial image anomaly detection. Inf. Fusion.

[2] Bergmann P., Fauser M., Sattlegger D., Steger C. MVTec AD—A comprehensive real-world dataset for unsupervised anomaly detection. Proceedings of the IEEE/CVF Conference on Computer Vision and Pattern Recognition (CVPR).

[3] Wang J., Niu Y., Huang B. (2025). Fusion-restoration model for industrial multimodal anomaly detection. Neurocomputing.

[4] Bergmann P., Jin X., Sattlegger D., Steger C. (2021). The MVTec 3D-AD dataset for unsupervised 3D anomaly detection and localization. arXiv.

[5] Wang Y., Peng J., Zhang J., Yi R., Wang Y., Wang C. Multimodal industrial anomaly detection via hybrid fusion. Proceedings of the IEEE/CVF Conference on Computer Vision and Pattern Recognition (CVPR).

[6] Cao Y., Xu X., Shen W. (2024). Complementary pseudo multimodal feature for point cloud anomaly detection. Pattern Recognit..

[7] Zou Y., Jeong J., Pemula L., Zhang D., Dabeer O. Spot-the-difference self-supervised pre-training for anomaly detection and segmentation. Proceedings of the European Conference on Computer Vision (ECCV).

[8] Zavrtanik V., Kristan M., Skočaj D. DRAEM: A discriminatively trained reconstruction embedding for surface anomaly detection. Proceedings of the IEEE/CVF International Conference on Computer Vision (ICCV).

[9] Liu Y., Zhuang C., Lu F. (2021). Unsupervised two-stage anomaly detection. arXiv.

[10] Wolleb J., Bieder F., Sandkühler R., Cattin P.C. Diffusion models for medical anomaly detection. Proceedings of the International Conference on Medical Image Computing and Computer-Assisted Intervention (MICCAI).

[11] Hoang D.-C., Tan P.X., Nguyen A.-N., Pham M.-K., Duong T.H.A., Huynh T.-M., Bui S.-A., Nguyen D.-M., Ha Q.-H., Trinh V.-A. (2025). Unsupervised industrial anomaly detection using paired well-lit and low-light images. J. Comput. Des. Eng..

[12] Gudovskiy D., Ishizaka S., Kozuka K. CFlow-AD: Real-time unsupervised anomaly detection with localization via conditional normalizing flows. Proceedings of the IEEE/CVF Winter Conference on Applications of Computer Vision (WACV).

[13] Rudolph M., Wehrbein T., Rosenhahn B., Wandt B. Fully convolutional cross-scale flows for image-based defect detection. Proceedings of the IEEE/CVF Winter Conference on Applications of Computer Vision (WACV).

[14] Rudolph M., Wandt B., Rosenhahn B. Same same but differnet: Semi-supervised defect detection with normalizing flows. Proceedings of the IEEE/CVF Winter Conference on Applications of Computer Vision (WACV).

[15] Bergmann P., Fauser M., Sattlegger D., Steger C. Uninformed students: Student-teacher anomaly detection with discriminative latent embeddings. Proceedings of the IEEE/CVF Conference on Computer Vision and Pattern Recognition (CVPR).

[16] Cao Y., Wan Q., Shen W., Gao L. (2022). Informative knowledge distillation for image anomaly segmentation. Knowl.-Based Syst..

[17] Tien T.D., Nguyen A.T., Tran N.H., Huy T.D., Duong S., Nguyen C.D.T., Truong S.Q.H. Revisiting reverse distillation for anomaly detection. Proceedings of the IEEE/CVF Conference on Computer Vision and Pattern Recognition (CVPR).

[18] Park K.-B., Lee J.Y. (2022). Novel industrial surface-defect detection using deep nested convolutional network with attention and guidance modules. J. Comput. Des. Eng..

[19] Ullah W., Khan S.U., Kim M.J., Hussain A., Munsif M., Lee M.Y., Seo D., Baik S.W. (2024). Industrial defective chips detection using deep convolutional neural network with inverse feature matching mechanism. J. Comput. Des. Eng..

[20] Chu Y.-M., Chieh L., Hsieh T.-I., Chen H.-T., Liu T.-L. Shape-guided dual-memory learning for 3D anomaly detection. Proceedings of the International Conference on Machine Learning.

[21] Wang C., Zhu H., Peng J., Wang Y., Yi R., Wu Y., Ma L., Zhang J. (2025). M3DM-NR: RGB-3D noisy-resistant industrial anomaly detection via multimodal denoising. IEEE Trans. Pattern Anal. Mach. Intell..

[22] Chen R., Xie G., Liu J., Wang J., Luo Z., Wang J., Zheng F. EasyNet: An easy network for 3D industrial anomaly detection. Proceedings of the 31st ACM International Conference on Multimedia (ACM MM).

[23] Bi C., Li Y., Luo H. Dual-branch reconstruction network for industrial anomaly detection with RGB-D data. Proceedings of the International Conference on Image, Signal Processing, and Pattern Recognition (ISPP 2024).

[24] Zavrtanik V., Kristan M., Skočaj D. Cheating depth: Enhancing 3D surface anomaly detection via depth simulation. Proceedings of the IEEE/CVF Winter Conference on Applications of Computer Vision (WACV).

[25] Liu J., Mou S., Gaw N., Wang Y. (2025). Uni-3DAD: GAN-inversion aided universal 3D anomaly detection on model-free products. Expert Syst. Appl..

[26] Rudolph M., Wehrbein T., Rosenhahn B., Wandt B. Asymmetric student-teacher networks for industrial anomaly detection. Proceedings of the IEEE/CVF Winter Conference on Applications of Computer Vision (WACV).

[27] Gu Z., Zhang J., Liu L., Chen X., Peng J., Gan Z., Jiang G., Shu A., Wang Y., Ma L. (2024). Rethinking reverse distillation for multi-modal anomaly detection. AAAI Conf. Artif. Intell. (AAAI).

[28] Cheng Y., Chen J., Wen G., Tan X., Liu X. (2025). LPFSTNet: A lightweight and parameter-free head attention-based student–teacher network for fast 3D industrial anomaly detection. Neurocomputing.

